# How the smart product attributes influence consumer adoption intention

**DOI:** 10.3389/fpsyg.2023.1090200

**Published:** 2023-02-23

**Authors:** Ming Li, Xuchen Bai, Saipeng Xing, Xueying Wang

**Affiliations:** ^1^School of Management, Wuhan Technology and Business University, Wuhan, Hubei, China; ^2^China Institute of Education and Social Development, Beijing Normal University, Beijing, China; ^3^Management School, Wuhan Technology and Business University, Hubei Business Service D&R Center, Wuhan, China

**Keywords:** smart product attributes, adoption intention, psychological empowerment, consumer innovativeness, connection attributes, intelligence attributes

## Abstract

Compared with traditional products, the connection attributes and intelligence attributes of the smart product are their differentiated competitive advantages. In order to understand how smart product attributes affect consumer attitudes and the influencing mechanism, we carry out this study. In the framework of psychological empowerment, this paper explores the relationship between smart product attributes and consumers’ adoption intention. We consider that companies can launch a range of smart products, where the probability of success is related to the degree to which intelligent and connection attributes stimulate consumer motivation. Smart products with intelligence attributes and connection attributes can improve consumers’ cognition of the four motivations consist of meaning, ability, autonomy and influence, which activate consumers’ psychological sense of empowerment, and thus improve consumers’ willingness to adopt. In addition, we also find that consumer heterogeneity influences this process. This paper mainly reports the moderating effect of Consumer domain-specific innovation. We find that the connection and intelligence attributes of smart products stimulate consumers’ adoption intention effectively. The findings of this paper complement innovation management literature related to smart product attributes and provide suggestions for enterprises to introduce smart products.

## Introduction

In the context of the development of the Internet of Things, artificial intelligence and other related technologies have been widely applied in products (Kozinets and Gretzel, 2021). By 2025, we predict that the scale of the global market of Internet of Things will swell to 1.6 trillion dollars (Tankovska, 2020). Moreover, smart products have become the hot topics, which have aroused the strong interest of academic researchers (Bstieler et al., 2018; Shim et al., 2019). Consequently, compared to the traditional products, what are the cardinal differences of the smart products attracting more attention? Whether these differences contribute to increase the consumer’s adoption intention?

Product attributes can reflect the differences of products, which are the collection of differentiated advantages as well (Mano and Oliver, 1993). Compared with traditional products, smart products are introduced connection components and intelligent components based the original physical components (Poter and Heppelmann, 2015). For example, traditional washing machines can only provide basic functions such as rinsing, while smart washing machines can also provide recommendations for using patterns by recognizing the condition of clothes based on its connection components and intelligence components. In other words, traditional products can only provide basic functions, while smart products can provide more functions and services in addition to basic functions (Yang et al., 2009). Therefore, we argue that, based on the functional changes brought by the attributes of smart products, different consumer experience is created, which can affect the change of consumers’ attitudes (Novak and Hoffman, 2019). What’s more, when purchasing products, product attributes are the primary factor for consumers to consider, where product attributes affect consumers’ evaluation of products and their purchase and adoption decisions (Ruby and Nikhilesh, 2004; Chitturi et al., 2008). Previous studies have defined the product attributes of smart products from different perspectives (Raff et al., 2020; Henkens et al., 2021), and recognized that the ownership of smart products is the key to distinguish them from traditional products (Yang et al., 2009). But we have found that they failed to comprehensively investigate the impact of product attributes of smart products on consumers’ adoption intention. Hence, we propose our research questions. How the attributes of smart products affect consumers’ adoption intention? And why the attributes have such an impact on adoption intention?

For researches about the new product adoption, we always consider the motivations of consumers (Davis, 1989). Therefore, we introduce psychological empowerment to measure the motivation, specially. The study of Nelson has proved that with the smart watch, consumer knows their health situation more and then his psychological empowerment increases (Nelson et al., 2016). Owing to their results, we continue to have an exploration on how the attributes of smart products affect the psychological empowerment and then the adoption intention. Besides, we acknowledge that the heterogeneity of consumer may impact. We take the Consumer domain-specific innovation into consideration as well.

This study therefore makes several theoretical contributions. First, this paper further divides and defines the attributes of smart products, and empirically tests the potential influence mechanism. The research results of this paper bridge the gap between smart product attributes and adoption intention. Second, we bridge the link between product attributes and adoption intention by including psychological empowerment (Spreitzer, 1995) and establishing that smart product attributes shape the psychological empowerment to increase the adoption intention. The results further prove that the psychological empowerment may affect individual adoption intention and behavior, expanding the application in the marketing field.

## Conceptual framework

### Theory

The theory of reasoned action presupposes that people are rational and have complete control over their actions. According to this theory, individual behavioral intention can be replaced by behavioral intention to some extent, and attitude and subjective behavioral norms also affect individual behavioral intention. The theory of reasoned action points out that information and motivation are two important factors that affect people’s behavior (Wu, 2021).

Actually, the uncertainty of new products is a key deterrent to adoption. As the essential characteristics of new products that distinguish them from other products, consumers can judge the advantages and risks of products based on product attributes, thus reducing the risk perception brought by information uncertainty and improving the willingness and behavior of adoption (Nelson, 1970). In other words, owing to the theory of reasoned action, product attributes provide important information to motivate consumers to take action. In addition, psychological empowerment reflects the individual’s cognition of motivation and can effectively motivate the individual to act rationally.

### Smart product attributes

Despite a growing body of literature on smart products, a consensus about what attributes constitute the smart products is lacking. To identify the attributes, we engaged in a review about what the smart products compose of. The smart product has three product modules, the physical module, the connection module, and the intelligent module (Poter and Heppelmann, 2015). With connection module and intelligence module, smart products derive capabilities different from traditional products, such as cooperation ability and learning ability (Rijsdijk and Hultink, 2009), and show unique advantages (Yang et al., 2009). Owing to this physical compound, we identified two attributes: connection attribute and intelligence attribute.

The one attribute of smart products is the connection attribute, which refers to the ability to connect with other participating objects in the product ecosystem, and the surrounding physical environment to obtain information (Verhoef et al., 2017; Pardo et al., 2020). This information is captured through the connection module (Rijsdijk and Hultink, 2009). Thus, the perception range for users expands (Henkens et al., 2021). For example, a smart lighting such as Philips Hue, is able to link with smart watches to get the information about the schedule, to get understanding of the variation of natural light.

The other attribute of smart products is the intelligence attribute, which refers to the ability to provide users with solutions based on independent learning and iterative optimization of computing logic (Rijsdijk and Hultink, 2009; Henkens et al., 2021; Rokonuzzaman et al., 2022). This information is captured through the intelligence module, the sensors and actuators part (Rijsdijk and Hultink, 2009; Pardo et al., 2020). For example, the driverless vehicles can learn the driving conditions to calculate the best autonomous driving solution.

Based on above two attributes of smart products, different levels of service emerge. Besides, the attributes and the emerging service provide consumers with optimized experience different from traditional products, which is the relative advantage of intelligent products (Rogers, 2003). To be honest, the smartness can vary from low (low levels of connection attribute and intelligence attribute) to high (high levels of connection attribute and intelligence attribute). Product attributes affect consumers’ evaluation of products and influence their purchase and adoption decisions (Ruby and Nikhilesh, 2004). These relative advantage sensing brought by the attributes of smart products are an important driving force for consumers to accept smart products (Taylor and Todd, 1995; Chitturi et al., 2008). As many product providers are improving the smart service of their offerings (Beverungen et al., 2019; Langley et al., 2020), we examine how the two attributes as the key differentiators compared to the traditional products influence consumer attitudes.

### Adoption intention

Adoption intention refers to the extent to which consumers are the first to use and accept new technologies, products or services compared to other members in the same social system context (Rogers, 2003). It means when consumer adopt the new product, he has taken a try and accepted it. Based on the diffusion theory of innovation, external factors have a significant impact on adoption intention, mainly including environmental factors and technological factors, and the relative perceived advantage of new technology (new product) is an important factor for adoption (Rogers, 2003).

At present, the research on the adoption intention of smart products is mainly elaborated from the perspective of motivation, combining TAM model and innovation diffusion theory. Through previous researches, we have known the influence of autonomous and other intelligence attributes of autonomous vehicles on adoption intention (Meyer and Cloarec, 2022). We have also known the influence of some other smart product attributes on consumer adoption and satisfaction (Rauschnabel et al., 2018; Henkens et al., 2021). It seems that the attributes of smart products have a positive impact on consumers’ adoption intention. In our context, we believe that the connection attribute and intelligence attribute will help to increase the adoption intention, because of the.

Compared with traditional products, in addition to basic functions, smart products can provide additional functions based on connection attributes and intelligence attributes (Yang et al., 2009), which can help consumers achieve behavioral goals and performance expectations (Meyer and Cloarec, 2022). In other words, the smart products have significant breakthrough innovation in connection attributes and intelligence attributes (Poter and Heppelmann, 2015). According to Roger’s findings (2003), the connection attributes and intelligence attributes are the advantages compared to the traditional products, which will have a positive impact on consumers’ adoption intention.

Specifically, smart products with the connection attribute can connect with more objects and physical environment (Verhoef et al., 2017). It expands the scope of information transmission, and increases the number of information exchange objects, which improves the timeliness and accuracy of information acquisition (Raff et al., 2020). For example, the ability to simultaneously interconnect with multiple users, to sense changes in temperature or light in the environment, or to obtain data needed from multiple cloud systems, can help consumers timely and accurately obtain various data, including data of themselves, connection objects and the environment. In other words, smart products with the connection attribute have obvious advantages in exchanging and transmitting information and improving consumers’ ability to obtain information, which brings obvious perception of relative advantage and usefulness to consumers and can improve their adoption intention (Taylor and Todd, 1995). Therefore, we hypothesize the impact of the connection attribute on adoption intention as follows:

**H1a.** The connection attribute of smart products is promoting consumers’ adoption intention.

Smart products with intelligence attributes can provide functions and services fitting (Henkens et al., 2021), assist consumers in making decisions and provide intelligent solutions (Rijsdijk and Hultink, 2009). For example, the abilities of booking tasks, navigation route planning, personalized recommendation, in line with the requirements of consumers, are providing convenience for consumers. In other words, smart products with the intelligence attribute have obvious advantages in providing personalized functions and improving consumers’ use efficiency, thus improve consumers’ living and working efficiency (Rauschnabel et al., 2018). Intelligence attribute brings obvious perception of comparative advantage and usefulness, which improves adoption intention (Taylor and Todd, 1995). Therefore, we hypothesize the impact of the intelligence attribute on adoption intention as follows:

**H1b.** The intelligence attribute of smart products is promoting consumers’ adoption intention.

### Psychological empowerment

As we demonstrate before, studies on adoption intention have mostly adopted a motivation perspective. To be more precise, the existing research is based on the theory of behavior, including the theory of reasoned action and the theory of planned behavior. Based on the theory of reasoned action and innovation diffusion theory, scholars have developed a series of research models, such as TAM and VAM. Therefore, we continue to follow the theory of reasoned action to explore the internal attitude factors of the theory of reasoned action consumers forming adoption intention because of the smart products attributes.

In particular, we introduce psychological empowerment to explore changes in consumer motivation. Psychological empowerment is defined as a motivational structure composed of four cognitions: meaning, ability, autonomy and influence, reflecting individuals’ positive orientation toward tasks (Spreitzer, 1995). Specifically, meaning refers to an individual’s cognition of positive outcomes, which is the belief that his behavior is valuable or important (Zhang and Bartol, 2010). Ability is the individual’s cognition of his own ability, who believes that it has the corresponding skills and knowledge to complete the behavior (Conger and Kanungo, 1988). Autonomy is an individual’s cognition of autonomous power, who believes that it can freely decide how to complete its behavior (Avolio et al., 2004). Influence refers to an individual’s cognition of the influence of action, who believes that his behavior has an impact on a certain target or a broader object (Spreitzer, 1995).

It’s acknowledged that confidence in ability, internal control points, information, feedback and other factors will affect the four kinds of cognition and have an impact on psychological empowerment, and thus affecting innovation-related behaviors (Spreitzer, 1995). As we have discussed before, product attributes as a signal, or information, can reduce consumer adoption barriers due to information uncertainty (Nelson, 1970). Hence, combining with the theory of reasoned action, we speculate that the smart product attributes, as the product information will also affect the internal motivation cognition of consumers’ sense of empowerment, thus generating positive attitudes and influencing consumers’ adoption intentions.

In the field of consumer behavior, this four-factor affecting mechanism still holds true. It has been proved that when consumers can control their access to product information and understand others’ comments on products timely, their sense of psychological empowerment is significantly increased (Wathieu and Brenner, 2002). A similar transmission mechanism is also shown in the context of smart products. The use of smart bracelets enables consumers to have a comprehensive and clear understanding of their health information and the process of health goals, which performs their advantages in obtaining information and feedback well, and improving psychological empowerment effectively (Nelson et al., 2016).

More importantly, based on the theory of reasoned action, psychological empowerment consisting can influence individual behavior. When the four cognition is met, the individual will get the experience of accumulation (Monje et al., 2021), and be stimulated to produce and continue the task behavior (Conger and Kanungo, 1988), and improve adoption intention. Hence, we suggest that the motivation structure of psychological empowerment can influence consumers’ adoption intention. Previous studies have backed it. The increase of consumers’ psychological empowerment will increase their willingness to interact with enterprises or other consumers (Ramani and Kumar, 2008). So they’re more excited to obtain more product information, which improves consumers’ evaluation of products and stimulates consumers’ purchase decisions (Ramani and Kumar, 2008; Broniarczyk and Griffin, 2014; Nelson et al., 2016).

In our context, we argue that with smart product attributes, psychological empowerment increases. The connection attribute expands the range of consumers to obtain information effectively, reduces the cost of consumers to collect information (Raff et al., 2020), improves the efficiency of information collection, and enhances the ability to obtain information (Henkens et al., 2021). It improves their evaluation of meaning and ability. In addition, due to the progress of connection technology, the connection behavior breaks the time limit, where consumers can take the connection behavior freely. They are able to cooperate with more objects and environments (Verhoef et al., 2017), which expands the scope of influence of consumer behavior and even influences enterprise behavior (Henkens et al., 2021). It improves their evaluation of influence. The connection attributes may also cause perceptual illusions of non-mediation (Hamilton and Yao, 2018), where the consumers believe that their behaviors result in the good performance rather than the excellent mediation. It improves their evaluation of autonomy. In summary, the connection attribute enhances the cognition of meaning, ability, autonomy and influence, thus the psychological empowerment increases, which leads to more adoption intention. Therefore, we hypothesize the mediating role of psychological empowerment between the connection attribute and the adoption intention as follows:

**H2a.** The psychological empowerment mediates the impact of connection attribute on adoption intention, where the positive effect of connection attribute through the psychological empowerment works on adoption intention.

The intelligence attribute of smart products assists consumers in making decisions (Raff et al., 2020), provides intelligent services for consumers, supplies consumers’ abilities (Blut et al., 2021), and helps them with achieving tasks or behavioral goals (Nelson et al., 2016). It improves their evaluation of ability. With intelligence attributes, the consumer’s unique needs are met, and the task efficiency is improved, which brings convenience and self-efficacy to consumers (Rauschnabel et al., 2018; Henkens et al., 2021). It improves their evaluation of meaning. In addition, due to the intelligence attribute, the iterative optimization function also provides suggestions to enterprises for product upgrading and influences enterprise behavior (Henkens et al., 2021; Rokonuzzaman et al., 2022). It improves their evaluation of influence. To sum up, the intelligence attribute enhances the cognition of meaning, ability, and influence, thus the psychological empowerment increases, which leads to more adoption intention. Therefore, we hypothesize the mediating role of psychological empowerment between the intelligence attribute and the adoption intention as follows:

**H2b.** The psychological empowerment mediates the impact of intelligence attribute on adoption intention, where the positive effect of intelligence attribute through the psychological empowerment works on adoption intention.

### Consumer domain-specific innovation

In addition to external environmental factors, consumers’ heterogeneity characteristics can also affect their acceptance of new products or technologies (Rogers, 2003). In relevant studies on innovation diffusion, consumer innovation has been constantly concerned and regarded as an internal force affecting innovation behavior (Roehrich, 2004). Consumer domain-specific innovation refers to the trend of consumers’ understanding and adoption of innovation (new product) in a specific field (Goldsmith and Hofacker, 1991), which can be used to measure the degree to which consumers are more likely to adopt a specific product category than others (Kaushik and Rahman, 2014).

Consumer domain-specific innovation is the key to predict willingness to adopt new products (Jeong et al., 2017). Consumers with high Consumer domain-specific innovation in the product category are able to contact and obtain information about such new products earlier and adopt such new products earlier than other consumers (Rokonuzzaman et al., 2022). They can bear the economic and mental loss caused by the failure of adoption decisions (Xie and An, 2020), and are more willing and easier to accept new products and technologies. With low Consumer domain-specific innovation, the result is opposite. Moreover, we have found the similar effect in the field of smart products (Hirunyawipada and Paswan, 2006; Jeong et al., 2017). Therefore, when studying the influence mechanism of smart product attributes on consumers’ willingness to adopt, we take the key individual trait of Consumer domain-specific innovation into consideration.

To be specific, consumers in high Consumer domain-specific innovation are willing to take the initiative to learn product knowledge, are more likely to pay attention to product information transmitted by connection attribution. They will have a better understanding of the advantages of smart products in information acquisition and transmission (Yi et al., 2006). In other words, the perception of the usefulness of the product is higher, their motivation to adopt is higher. Hence, they are more likely to form positive cognition of motivation, such as ability and meaning, and thus have a higher level of psychological empowerment. However, consumers with low Consumer domain-specific innovation are less willing to take the initiative to learn and pay attention to product knowledge, have less obvious perception of advantages of connection attributes. What’s more, they are more likely to worry about negative effects such as privacy invasion (Henkens et al., 2021), which results in the negative cognition of meaning and influence, and the lower level of psychological empowerment. Therefore, we hypothesize the moderating role of Consumer domain-specific innovation as follows:

**H3a.** Consumer domain-specific innovation positively moderates the relationship between connection attribute and psychological empowerment.

Similar to connection attribute, consumers with high Consumer domain-specific innovation have a better understanding of the advantages of intelligence attribute in fitting personalized needs and improving efficiency. They are more likely to pay attention to the relative advantages of intelligence attribute (Yi et al., 2006), and have a more positive recognition of ability and meaning, with the higher psychological empowerment. However, consumers with low Consumer domain-specific innovation have less understanding of the advantages of intelligence attributes, and are more likely to perceive autonomy conflicts due to intelligence attributes (Jeong et al., 2017; Wang, 2021), resulting in a lower level of psychological empowerment. Therefore, we hypothesize the moderating role of Consumer domain-specific innovation as follows:

**H3b.** Consumer domain-specific innovation positively moderates the relationship between intelligence attribute and psychological empowerment.

**FIGURE 1:**
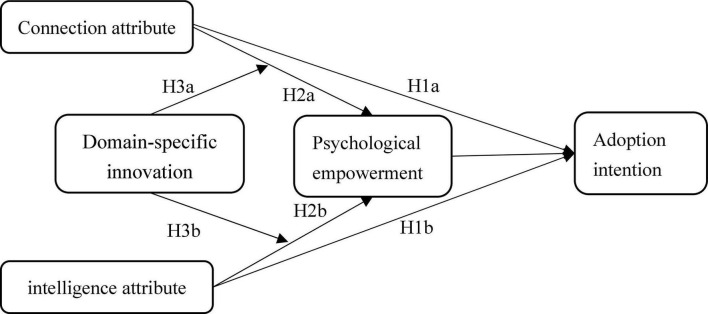
The model diagram.

To sum up, our model diagram is shown in Figure 1.

## Materials and methods

### Subjects and procedure

The study’s setting is China, where the artificial intelligence industry is developing well (Iimedia, 2020). We mainly recruited survey subjects through the Credamo platform and completed the questionnaire. Credamo, is a professional level of online research service platform website. In previous studies, scholars have conducted questionnaires on this platform and obtained reliable research results. Credamo invites users to fill in the questionnaire by randomly sending them a hyperlink to the questionnaire. Since it may be difficult to control the high concentration of the respondents by filling in the questionnaire online, we set the attention screening item as Smith et al. (2016). “Please select 2 for this question.” After passing the attention screening, each participant can receive a 5-yuan platform red envelope. To ensure each participant must have been experienced the smart product, we asked subjects to recall and write down the recent smart products they used first. Finally, the 204 study participants, invited by the questionnaire research platform, vary in age from 18 to 35 years (mean age = 22.4 years). On average, participants (52.9% female, 94% between 19 and 25 years of age) had experienced at least 3 different types of smart products.

At the beginning of the study, participants first wrote down the smart products they had experienced and described how it felt. The most frequently mentioned (more than 90%) were smart phones and smart watches. Participants imagined that they had employed the “smart products that you are familiar with and engage in your regular usage activities.” This survey was administered online in Chinese and followed standard back-translation procedures (Brislin, 1976).

### Measures

We develop the survey instrument by adopting existing validated scales when possible. On the basis of the pretest, we also refine the questionnaire and modify some items. We present the constructs and item details in Table 1.

**TABLE 1:** Confirmatory factor analysis of the measures.

Construct and source	Operational measures of construct	SFL
Model fit indexes *X*^2^ 1013.28, *df* = 485, *X*^2^*/df* = 2.09; GFI = 0.80, CFI = 0.90, RMSEA = 0.05		
Connection attribute AVE = 0.50 CR = 0.70	1. This smart product is connected to different actors.	0.87
	2. This smart product can communicate with different actors.	0.80
	3. This smart product can cooperate with different actors.	0.87
	4. This smart product keeps an eye on itself and its environment.	0.86
	5. This smart product observes itself and its environment.	0.90
	6. This smart product is aware of itself and its environment.	0.89
Intelligence attribute AVE = 0.52 CR = 0.80	1. This smart product takes previous collected information into account to make decisions.	0.85
	2. This smart product can improve itself by learning.	0.82
	3. This smart product can do things by itself.	0.85
	4. This smart product can work independently.	0.88
	5. This smart product can take initiative.	0.88
	6. This smart product can go its own way.	0.81
Adoption intention AVE = 0.56 CR = 0.79	1. I am happy to use this smart product.	0.85
	2. I will use this smart product.	0.86
	3. I would recommend this smart product to people around me.	0.82
Psychological empowerment AVE = 0.51 CR = 0.84	1. Using this smart product is meaningful to me.	0.67
	2. My using activities are personally meaningful to me.	0.67
	3. Using this smart product is very important to me.	0.59
	4. I am confident about my ability to use this smart product.	0.67
	5. I am self-assured about my capabilities to perform my using activities.	0.61
	6. I have mastered the skills necessary for this smart product.	0.76
	7. I have significant autonomy in determining how I use this smart product.	0.79
	8. I can decide on my own how to use this product.	0.68
	9. I have considerable opportunity for independence and freedom in how I use this product.	0.85
	10. My impact on what happens in my product ecosystem is large.	0.86
	11. I have a great deal of control over what happens in my product ecosystem.	0.92
	12. I have significant influence over what happens in my product ecosystem.	0.91
Consumer domain-specific innovation AVE = 0.58 CR = 0.84	1. I am the first of my friends to buy when the new smart products came on the market.	0.73
	2. I have more smart products than my friends.	0.65
	3. I would like to buy new smart products even without a trial.	0.61
	4. I like to buy new smart products before others.	0.77
	5. If there is a new smart product on the market, I will be happy to buy it.	0.77
	6. I am the first to know the name of the new smart product.	0.81

SFL, standardized factor loading; AVE, average variance extracted; CR, composite reliability; df, degrees of freedom; GFI, goodness-of-fit index; CFI, confirmatory fit index; RMSEA, root mean square error of approximation. The scale format for each of these measures is 1 = “strongly disagree” and 5 = “strongly agree”.

Our conceptualization of “smart product attributes” is based on Poter and Henkens’s work (Poter and Heppelmann, 2015; Henkens et al., 2021) and consists of two dimensions: the connection attribute and the intelligence attribute. We adapt the connection attribute construct from Rijsdijk and Henkens’s work (Rijsdijk and Hultink, 2009; Henkens et al., 2021). It consists of six items with which we examine the extent to which the users feel that the smart products connect the physical environment and participants through the Internet of Things, enhancing the ability to expand the scope of perception. We adapt the intelligence attribute construct from the same sources (Rijsdijk and Hultink, 2009; Henkens et al., 2021). It consists of six items with which we examine the extent to which the users feel that the smart products independently learn and iteratively based on computing logic to provide solutions for users.

The adoption intention norms, from Dodds’s work, refer to the willingness of consumers to adopt smart products (Dodds et al., 1991). We measure psychological empowerment with twelve items from Spreitzer (1995), which reveals the extent to which the individual has the cognition of meaning, ability, autonomy, and influence, the positive orientation to the task. Finally, we leverage six items to measure Consumer domain-specific innovation on the basis of Roehrich’s (2004) conceptualization and thus examine personal characteristics affecting differences in attitudes and behavior of smart products. All items use seven-point Likert scales (1 = strongly disagree, 5 = strongly agree).

After data collection, we first test the reliability and validity to prove the reliability and credibility of the data collection results. Next, we use linear regression analysis to test our hypothesis, and report the results of the bootstrap.

## Results

### Reliability and validity

To test the validity and reliability of our study’s measurement model, we exploit the confirmatory factor analysis (CFA). The CFA model includes connection attribute, intelligence attribute, adoption intention, psychological empowerment and Consumer domain-specific innovation. The results indicate that the CFA model offer acceptable fit [χ^2^ = 1013.28, degrees of freedom (df) = 485, χ^2^/*df* = 2.09, goodness-of-fit index (GFI) = 0.80, confirmatory fit index (CFI) = 0.90, root mean squared error of approximation (RMSEA) = 0.05].

To estimate the internal consistency of the constructs, we take advantage of composite reliability (CR) and average variance extracted (AVE). The CR for all constructs are greater than the 0.7 threshold (Hair et al., 1998), and the AVE are more than the recommended 0.5 level (Hair et al., 1998). It means over half of the variance observed is accounted for by the hypothesized constructs. In addition, owing that the CR and AVE for all constructs in the model (see Table 1) are significantly higher than the stipulated criteria, the internal consistency is good.

To assess convergent validity, we use the factor loading matrix. All items load on the expected structures without significant cross-loading terms, which supports the convergent validity of the measurement items and the unidimensionality of the latent constructs (Steenkamp and van Trijp, 1991).

To test the discriminant validity, we compare the Pearson correlation coefficients between the two latent constructs with the square root of the AVE for each construct (see Table 2). And we find that under any circumstances, the Pearson correlation coefficients is lower than the square root of the AVE, which supports the discriminant validity of all constructs (Fornell and David, 1981).

**TABLE 2:** Construct correlation values and comparison with average variance extracted (AVE).

	I	II	III	IV	V
I. Connection attribute	(0.71)				
II. Intelligence attribute	0.43	(0.72)			
III. Adoption intention	0.40	0.21	(0.75)		
IV. Psychological empowerment	0.62	0.50	0.61	(0.71)	
V. Consumer domain-specific innovation	−0.35	−0.33	−0.73	−0.46	(0.76)

Numbers in parentheses are the square root of each AVE value.

To sum up, the internal consistency, convergent validity, and discriminant validity perform well, and we may proceed to estimate the model.

Because in the form of a questionnaire, the results were filled out by the respondents themselves. In order to judge the existence of the homologous variance, we use the Harman one-way variance detection. The results show that the characteristic root is greater than 1 without the rotation, the maximum factor contribution rate is only 28.56%, less than 40%. Therefore, the data have no homologous variance problem.

### Linear regression analysis

We use linear regression analysis to test our hypothesis. The regression analysis results are shown in Table 3. The variance expansion factors of all models are less than 10, so there is no collinearity problem in the models. The model 1 only contains the controls, and the model 4 contains all the controls and independent variables. According to model 2, connection attribute has a significant positive impact on adoption intention (β = 0.41, *P* < 0.01), where H1a is supported. According to model 3, intelligence attribute has a significant positive influence on adoption intention (β = 0.15, *P* < 0.01), in support of H1b.

**TABLE 3:** Main-effect regression results.

Model	(1)	(2)	(3)	(4)
Connection attribute		0.41*** (0.07)		0.38*** (0.08)
Intelligence attribute			0.15*** (0.05)	0.03* (0.06)
Controls:				
Gender	0.14 (0.09)	0.14* (0.07)	0.16** (0.08)	0.15* (0.08)
Age	0.04 (0.84)	0.09 (0.17)	0.03 (0.18)	0.09 (0.17)
Education	0.13 (0.08)	0.14** (0.07)	0.11 (0.07)	0.13* (0.05)
Incomes	−0.03 (0.60)	−0.04 (0.05)	−0.04 (0.05)	−0.04 (0.05)
Constant	3.77*** (0.43)	2.06*** (0.49)	3.29*** (0.45)	2.05*** (0.49)
N	204	204	204	204
R	0.16	0.42	0.26	0.42
R2	0.03	0.17	0.07	0.17
△F	1.406	8.23***	2.9**	6.88***

***p < 0.01, **p < 0.05, *p < 0.1.

Hierarchical regression is used to analyze the mediating effect of psychological empowerment, where the results are shown in Table 4. The variance expansion factors of all models are less than 10, so there is no collinearity problem in the models. According to model 5, connection attribute had a significant positive effect on psychological empowerment (β = 0.49, *P* < 0.01). In model 6, after introducing the mediating variable psychological empowerment on the basis of Model 1, psychological empowerment has a significant positive effect on adoption intention (β = 0.72, *P* < 0.01), and the regression coefficient of connection attribute on adoption intention is not significant (β = 0.02, *P* > 0.05). *R*^2^ rises from 0.36 to 0.41.

**TABLE 4:** Mediator effect regression results.

Model	(5)	(6)	(7)	(8)
**Independent variable**	**Psychological empowerment**	**Adoption intention**	**Psychological empowerment**	**Adoption intention**
Connection attribute	0.49*** (0.04)	0.02 (0.07)		
Intelligence attribute			0.32*** (0.04)	−0.11** (0.05)
Psychological empowerment		0.72*** (0.09)		0.82*** (0.08)
Controls:				
Gender	0.01 (0.06)	0.13** (0.07)	0.06 (0.06)	0.12* (0.06)
Age	0.07 (0.12)	0.04 (0.14)	−0.01 (0.13)	0.04 (0.14)
Education	0.01 (0.05)	0.14** (0.06)	−0.05 (0.06)	0.15** (0.06)
Incomes	0.01 (0.03)	−0.05 (0.04)	−0.01 (0.04)	−0.04 (0.04)
Constant	1.85*** (0.36)	0.95** (0.42)	3.32*** (0.31)	0.74* (0.42)
N	204	204	204	204
R	0.60	0.64	0.50	0.65
R^2^	0.36	0.41	0.25	0.42
△F	22.31***	22.45***	13.20***	23.89***

***p < 0.01, **p < 0.05, *p < 0.1.

Then, we use model 4 in bootstrap to verify it. The result shows that the confidence interval for the indirect effect rises from 0.2417 to 0.5441 (β = 0.3826, *SE* = 0.0770), and the direct effect of connection attributes on adoption rises from −0.1317 to 0.1571 (β = 0.0127, *P* > 0.05). Thus, psychological empowerment plays a complete mediating role in connectivity and adoption willingness, assuming that H2a is supported. When exposed to connection attributes, the consumer feels positive psychological empowerment, which results in adoption intention.

According to model 7, intelligence attribute has a significant positive influence on psychological empowerment (β = 0.32, *P* < 0.01). In model 8, after introducing the mediating variable psychological empowerment on the basis of model 3, psychological empowerment has a significant positive effect on adoption intention (β = 0.82, *P* < 0.01), and the regression coefficient of intelligence attribute on adoption intention remains significant (β = −0.11, *P* < 0.05). *R*^2^ increases from 0.25 to 0.42.

Then, we use model 4 in bootstrap to verify it. The result shows that the confidence interval for the indirect effect rises from 0.1673 to 0.3484 (β = 0.2502, *SE* = 0.0459), while the direct effect of intelligence attributes on adoption rises from −0.1982 to −0.0131 (β = −0.1057, *P* < 0.05). Thus, psychological empowerment does not mediate the influence of intelligence attribute and adoption intention, and H2b is not supported.

Though we have verified that higher intelligence attribute leads to higher adoption intention, when we control the psychological empowerment, intelligence attributes have an insignificant negative impact on the adoption intention, which isn’t consistent with our former hypothesis. It means when exposed to intelligence attributes, the lower level of intelligence attributes will lead to higher psychological empowerment, and thus the adoption intention of the smart products. We will explain it in the discussion.

We also use hierarchical regression method to analyze the moderating effect of Consumer domain-specific innovation. The variance expansion factors of all models are less than 10, so there is no collinearity problem in the models. The results of the regulatory effect are shown in Table 5. Model 9 and Model 10 tests the moderating effect of Consumer domain-specific innovation on connection attribute and psychological empowerment. Model 11 and Model 12 tests the moderating effect of Consumer domain-specific innovation on intelligence attribute and psychological empowerment.

**TABLE 5:** Moderating effect regression results.

Model	(9)	(10)	(11)	(12)
**Independent variable**	**Psychological empowerment**	**Psychological empowerment**	**Psychological empowerment**	**Psychological empowerment**
Connection attribute	0.44*** (0.05)	0.05 (0.15)		
Intelligence attribute			0.24*** (0.04)	−0.15 (0.13)
Consumer domain-specific innovation	−0.24*** (0.04)	−0.91*** (0.24)	−0.26*** (0.05)	−0.86*** (0.20)
Connection attribute*Consumer domain-specific innovation		0.17** (0.06)		
Intelligence attribute*Consumer domain-specific innovation				0.18** (0.06)
Controls:				
Gender	−0.04 (0.05)	−0.04 (0.05)	−0.01 (0.06)	−0.02 (0.06)
Age	0.02 (0.11)	0.03 (0.11)	−0.05 (0.12)	−0.09 (0.12)
Education	−0.03 (0.05)	−0.04 (0.05)	−0.08 (0.05)	−0.09 (0.05)
Incomes	0.03 (0.03)	0.02 (0.03)	0.02 (0.04)	0.02 (0.03)
Constant	2.95*** (0.39)	4.48*** (0.67)	4.14*** (0.37)	4.98*** (0.70)
N	204	204	204	204
R	0.67	0.68	0.59	0.62
R^2^	0.45	0.47	0.35	0.38
△F	26.34***	24.53**	17.74***	17.30**

***p < 0.01, **p < 0.05, *p < 0.1.

On the basis of Model 9, the interaction term of Consumer domain-specific innovation and connection attribute has a significant positive effect on psychological empowerment (β = 0.17, *P* < 0.05), and the change of F was significant. What’s more, we use model 7 to verify the moderating effect. The result shows that the confidence interval for the indirect effect is from 0.0279 to 0.2122 (β = 0.1224, *SE* = 0.0607), in support of moderating mediating effect. It means that when the Consumer domain-specific innovation is high, the consumer is more willing to adopt the smart products with connection attributes, because of the psychological empowerment. Thus, H3a is supported.

**FIGURE 2:**
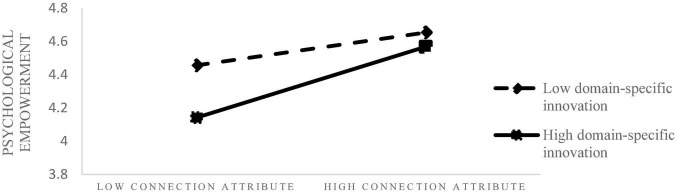
The moderating role of Consumer domain-specific innovation between connection attribute and psychological empowerment.

Under the condition of high level of consumer innovation in specific fields, consumers have a more inclusive attitude toward connection attributes, more expectations and positive motivation perception, thus bringing higher willingness to adopt. Figure 2 shows the moderating effect of Consumer domain-specific innovation on connection attribute and psychological empowerment.

On the basis of Model 11, after adding the interaction term between Consumer domain-specific innovation and intelligence attribute, model 10 shows a significant positive impact on psychological empowerment (β = 0.18, *P* < 0.05), and the change of F is significant. We also use model 7 to verify the moderating effect. The result shows that the confidence interval for the indirect effect is from 0.0095 to 0.2977 (β = 0.1362, *SE* = 0.0745), in support of moderating mediating effect. Hence, we find that, when the Consumer domain-specific innovation is high, he is more willing to adopt the smart products with intelligence attributes, because of the psychological empowerment. It supports H3b.

**FIGURE 3:**
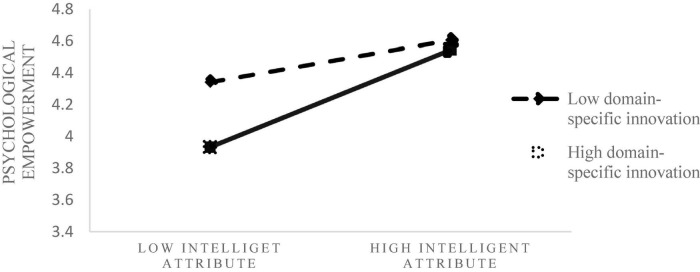
The moderating role of Consumer domain-specific innovation between intelligence attribute and psychological empowerment.

Under the condition of high level of consumer innovation in specific fields, consumers have a more inclusive attitude toward intelligence attributes, more expectations and positive motivation perception, thus bringing higher willingness to adopt. Figure 3 shows the moderating effect of Consumer domain-specific innovation on intelligence attribute and psychological empowerment.

## Discussion

The smart product has been the hot trend, and the market size is enlarging (Gartner, 2020; Vailshery, 2021). Previous researches based on Poter and Heppelmann’s (2015) achievements. Scholars have elaborated their understanding of the property of smart products from different angles (Rijsdijk and Hultink, 2009; Verhoef et al., 2017; Pardo et al., 2020; Henkens et al., 2021). But these studies sometimes fail to distinguish between the different product attributes brought about by different components. For example, Henkens has found out that the smart products have awareness attributes, connection attributes, self-determination attributes and dynamic attributes (Henkens et al., 2021). While compared to Poter and Heppelmann’s (2015), there are significant differences between connected and intelligent components. In other words, they have different physical basis. So, we cannot consider connection attributes as the part of intelligence attributes. That is why we speculate smart products to have two attributes, the connection attributes and the intelligence attributes.

To have a better understanding of smart products and explore the essential differences between it and traditional products, we investigate how two key attributes, the connection attribute and intelligence attribute, may influence consumer responses to adoption. Using a survey of smart products users, we find that connection attribute affects psychological empowerment, which could lead to more positive attitude toward adoption intention. Henkens’s research came to similar conclusions (2021). They have found that the connection attribute can influence consumer participation and thus consumer self-efficacy. It is not difficult to find that in this study, the connection attribute of smart products activates a kind of motivation perception of consumers, the self-efficacy. Along with this finding, we further explore the impact of connection attributes of smart products on the four-dimensional motivational structure of psychological empowerment, and thus on consumers’ adoption intention.

As for intelligence attribute, we fail to find the evidence to support that it results in psychological empowerment and thus adoption intention. Though we have verified that higher intelligence attribute leads to higher adoption intention, when controlling the psychological empowerment, intelligence attributes have an insignificant negative impact on the adoption intention, which isn’t consistent with our former hypothesis. Owing to this, we make the following explanations. First, the smart products with the different levels of intelligence attribute are in great difference. For example, smart watches are with low intelligence attributes, while smart robots are with high intelligence attributes. The latter has a negative impact on autonomy and ability evaluation (Wang, 2021), which affects the assessment of psychological empowerment, and affects consumers’ willingness to adopt. Second, there may be some other mediators affecting, such as privacy concerns (Henkens et al., 2021). While in our contexts, our questionnaire did not precisely define smart products, and the subjects mostly recalled smart products with weak smart attributes such as smart phones and smart bracelets, we could not effectively control the respondents to recall a consistent smart product use experience, and the privacy concerns are not significant. Third, according to the Uncanny Valley effect, the smarter the product, the more likely it is to be perceived as anthropomorphic, and the resistance is more obvious (Kim and Sundar, 2012). Therefore, when activating consumers’ motivation cognition, the high intelligence attributes of smart products may adversely affect consumers’ adoption intention.

The results of the moderating effect test in this paper indicate that Consumer domain-specific innovation has a moderating effect on psychological empowerment. Consumers with high Consumer domain-specific innovation are more interested in smart products, more inclusive, more willing to try smart products, and more confident in their own abilities. In other words, consumers with high Consumer domain-specific innovation perceive themselves as capable of using smart products and are more excited about them, increasing their sense of psychological empowerment. Low-innovation consumers, on the contrary, have a lower sense of psychological empowerment.

### Theoretical implications

We discuss some key study insights and their implications next. First, we add to the literature on innovation management related to smart product attributes based on an empirical study. Owing to the modular composition of smart products, we define the product attributes of smart products as connection attribute and intelligence attribute, which are the main advantages that distinguish them from traditional products. In the definition of connection attribute, in addition to the ability to connect and interact with other objects, we add the ability to connect with the physical environment itself, which broadens the scope of the definition of connection attribute of smart products. In the definition of intelligence attribute, in addition to the previous study of autonomous capability, the ability of smart products to learn and optimize themselves through iteration is also added to the original study, adding the iterative nature of smart products to the original study. We also contribute to the literature on the effect of smart product attributes on consumer adoption intentions. We empirically examine the impact of the attributes of smart products themselves, especially the functional attributes of connectivity and smartness, on product adoption intentions.

What’s more, though psychological empowerment is mostly used in human resource management, we incorporate the psychological empowerment element into innovation management research. The results of the mediation test show that increased connection attribute positively affects consumers’ sense of psychological empowerment and positively influences adoption intention, while increased intelligence attribute fail to positively affect psychological empowerment and thus the adoption intention. Increased intelligence reduces consumers’ sense of control and leads to conflicts over autonomy. This point warrants further research.

### Implications for practice

We make some implications for management as well. Enterprises can enhance consumers’ willingness to adopt smart products by increasing the product attributes. Companies can create smart products that are more perceptive of the physical environment and have a wider range of connected objects, and present this perceptiveness of the physical environment and range of connected objects to consumers intuitively to increase their willingness to adopt smart products. At the same time, enterprises also need to pay attention to the presentation of intelligent factors, including smart products to collect information, make decisions, and self-optimization. They can consider to increase the comprehensibility of this intelligent computing process, so that consumers have a clearer perception of the intelligent role of smart products, improve their perception of the advantages of smart products, and enhance their willingness to adopt them.

Also, by enhancing the connection attribute and displaying, companies can improve consumers’ ability to collect and control information, increase their sense of psychological empowerment, to optimize their experience of using the products, and enhance their willingness to adopt them. At the same time, companies should avoid the negative experience of consumer autonomy caused by the high level of intelligence attribute, and avoid reducing the positive experience of consumers and creating negative effects.

Consumer domain-specific innovation is an important personal characteristic for consumers to adopt smart products. Companies can identify target consumers with higher Consumer domain-specific innovation through their past shopping experiences, and transform these target consumers into early adopters of smart products, and then penetrate to more mid- to late-adopters for effective smart product diffusion.

### Limitations

We must admit that there are some shortcomings in this paper, which also provide clues for future research.

First, since the sample is mostly composed of young people, who are more willing to adopt new products and more tolerant of innovation failure than other age groups, so the randomness of the sample is not enough, which may interfere with the research results. While, we must admit that young people are the main force of consumers of smart products. Take our smart home market as an example, based on the investigation of INSIGHT AND INFO, the consumption of young people accounts for 87.2%, and they form the main consumption force in the market. Therefore, more reasonable sampling methods, such as stratified sampling, can be adopted in future studies to reduce sample bias among users.

Second, we use a questionnaire to measure the maturity of the respondents, which may interfere with the results and overly remind the respondents. Meanwhile, we begin with “please recall your recent experience with smart products,” but the respondents mostly recall smart products such as smart phones, ipads, and bracelets, but not other industries and other products, which may cause the generalization and measurement of the attributes of smart products to be not deep and comprehensive.

Third, we adopt cross-sectional data to analyze adoption intention, and not address long-term changes in consumer attitudes and behaviors. For real consumers, they may have different requirements for product attributes at different stages of product purchase decisions, and this dynamic change is not addressed in this study.

Fourth, there are differences in the smart products themselves. There are different types of smart products, and there are also significant differences between the same attributes (Li et al., 2017; Henkens et al., 2021). We did not investigate the detailed classification of intelligent products, and the lack of in-depth and detailed descriptions of the performance of the attributes of high and low levels may interfere with the results.

### Future work

In the face of the hot trend of artificial intelligence and smart products, we explore the influence of product attributes on adoption intention, and the attributes of smart products can be explored from more perspectives in the future.

First, more industries and categories of smart products can be included in the study to further improve the definition of connectivity and intelligence of smart products and provide more empirical test bases. At the same time, there are various ways to classify product attributes, such as functional attributes and enjoyment attributes (Okazaki et al., 2010). And there are also various other attributes of smart products, such as anthropomorphism (Rijsdijk and Hultink, 2009), which can be explored to investigate the impact of various product attributes of smart products on consumer adoption intention.

Second, we focus on the advantages of smart product attributes, but in reality, smart product attributes can also bring some negative effects. For example, smart attributes can lead to conflicts over autonomy (Wang, 2021), and connection attribute can lead to privacy concerns (Henkens et al., 2021). In future research, we suggest that, the negative effects of smart product attributes can be investigated to further guide enterprises to be alert to the negative effects and guide their practice. It is also possible to examine smart product attributes in a comprehensive manner and explore how consumers weigh and pay attention to the advantages and disadvantages of smart product attributes and their corresponding behaviors, so as to report the inner psychological trade-off process of consumers and improve related research.

Third, based on the investigation of the impact of smart product attributes on adoption intentions, we can further explore the impact of these two smart product attributes on consumer behavioral tendencies such as product evaluation and word-of-mouth communication.

Fourth, the empirical results of this paper indicate that the mediating role of consumers’ psychological empowerment in the path of the influence mechanism between intelligence attribute and adoption intention is not significant. Further research could investigate the generality and validity of this finding, whether it exists other essential mediators to explain the result. In addition, it is also possible to investigate whether there are mechanisms by which smart products with high and low intelligence attributes interact with feelings of psychological empowerment that have a significant impact on consumer attitudes.

Fifth, in addition to the factors of Consumer domain-specific innovation, future study can explore whether there are other personal attributes and external environmental stimuli that profoundly influence consumer adoption intentions, such as product usage scenarios. It may provide new ways of thinking about how to improve consumer adoption intention.

## Data availability statement

The raw data supporting the conclusions of this article will be made available by the authors, without undue reservation.

## Author contributions

ML and SX took responsibility to the conceptualization of the framework and theoretical foundation parts. XB and XW took responsibility to the experimental design, data collection, data analysis, and manuscript writing parts. All authors contributed to the article and approved the submitted version.
